# THE ROLE OF AGE AND EMOTION REGULATION IN CHOICE OF EMOTIONAL ACTIVITY IN EVERYDAY LIFE

**DOI:** 10.1093/geroni/igz038.2995

**Published:** 2019-11-08

**Authors:** Majse Lind, Derek Isaacowitz

**Affiliations:** Northeastern University, Boston, Massachusetts, United States

## Abstract

Situation selection is a form of activity selection focused on the affective tone of potential choices. We investigated this type of emotional activity selection in everyday life during a longitudinal multi-burst study of middle-aged and older adults. In each burst, participants were asked to complete six phone-based assessments per day across five consecutive days, including a situation selection question about what type of emotional situation they would want to engage in at the time. They also reported on various emotion regulation strategies. Both age groups favored positive activities, but older adults preferred positive-deactivated activities whereas middle-aged adults favored positive-activated activities. Both middle-aged and older adults reported more attention towards positive relative to negative thoughts. Greater attention to positive thoughts was associated with the tendency to select more positive activities across age groups. Both age and emotion regulation strategy use may therefore contribute to choice of emotional activities in everyday life.

